# Trial registration of abstracts from the American Society of Anesthesiologists Meetings 2010–2016: A review of prospective trial registration and selective outcome reporting

**DOI:** 10.1371/journal.pone.0270841

**Published:** 2022-07-05

**Authors:** Simon W. Chong, Georgina Imberger, Amalia Karahalios, Andrew Wang, Millicent Burggraf, Maleck Louis, Grace M. Liskaser, Anthony Bianco, Philip J. Peyton

**Affiliations:** 1 Department of Critical Care, The University of Melbourne, Melbourne, Australia; 2 Department of Anaesthesia, Pain and Perioperative Medicine, Western Health, Melbourne, Australia; 3 Centre for Epidemiology and Biostatistics, Melbourne School of Population and Global Health, The University of Melbourne, Melbourne, Australia; 4 Department of Anaesthesia, Austin Health, Melbourne, Australia; 5 Department of Surgery, Austin Health, Melbourne, Australia; Seoul National University College of Medicine, REPUBLIC OF KOREA

## Abstract

Mandatory prospective trial registration was introduced in 2005 to reduce publication bias and selective outcome reporting. In this study, we measured the proportion of prospective trial registration in randomized controlled trials in the anesthesia literature after this introduction, discrepancies between these trial protocols and subsequent publications, the association between being prospectively registered and reporting positive or negative results, and between being prospectively registered and achieving publication. We reviewed all abstracts from the American Society of Anesthesiologists annual meetings between 2010–2016 and included randomized controlled trials in humans. The abstract conclusions were scored as positive or negative according to predetermined definitions. We conducted a systematic search for trial registration and subsequent publication. Of the 9789 abstracts reviewed, 1070 abstracts were included. 222 (21%) of these abstracts had undergone prospective trial registration. 168/222 (76%) had a corresponding journal publication. 81(48%) had a major discrepancy between registration and publication. 149 (67%) of the abstracts with registration had positive outcomes compared with 616 (73%) of those without (Odds Ratio 0.77; 95% CI: 0.56 to 1.06; P = 0.105). Abstracts that had been registered were more likely to proceed to publication than those that had not (Odds Ratio 3.82; 95% CI 2.73 to 5.35; P < 0.001). The proportion of randomized controlled trials being prospectively registered in anesthesia remains low. Discrepancies between registry entries and corresponding journal publications are common. There was no association between prospective trial registration and subsequent positive outcomes. There was a strong association between prospective trial registration and the likelihood of progression to journal publication.

## Introduction

Positive publication bias occurs when studies with positive results are selectively submitted and accepted for publication over those with negative results [1]. Selective outcome reporting, the selection for publication of only a subset of outcomes from those that were planned, is inherently related to publication bias [2]. Publication bias and selective outcome reporting are significant issues in medical research [3–10], with Glasziou et al. noting in their Lancet Reward campaign publication that “unless research is adequately reported, the time and resources invested in the conduct of research is wasted” [11].

In 2005, the International Committee of Medical Journal Editors (ICMJE), on behalf of the major medical journals, attempted to address the issues of publication bias and selective outcome reporting by making prospective registration of clinical trials mandatory [12], stating that ICMJE journals would consider trials beginning on or after July 1, 2005 only if registration occurred before the first patient was enrolled. Unfortunately, despite this statement, many smaller journals have continued to publish randomized controlled trials (RCTs) that have not been prospectively registered [13].

When studies are prospectively registered, there are still often major discrepancies between protocols and the corresponding published papers [3,4,6,10,14]. Discrepancies include trial registration occurring retrospectively and changes to primary outcomes, sample sizes, and study interventions. In the anesthesia literature, two reviews have revealed very low rates of prospective trial registration in RCTs published in high impact journals and high rates of discrepancies between journal publications and corresponding trial registry entries [15,16].

Our study expanded on this previous work, using the abstracts presented at the American Society of Anesthesiologists (ASA) annual meetings, being the largest annual worldwide educational event in anesthesiology, as our ‘population’ of studies. This allowed inclusion of studies outside high impact journals and a broader understanding of how bodies of evidence are being affected by publication bias and selective outcome reporting. We measured the proportion of prospective trial registration in RCT abstracts presented, and assessed whether being registered was associated with a subsequent published trial result being positive or negative. Our hypothesis was that abstracts that were not prospectively registered were more likely to report a positive outcome in a subsequent publication. In addition, we also sought to quantify the proportion of major discrepancies between abstract trial registration entries and their corresponding journal publications.

## Methods

This study was prospectively registered and the protocol uploaded on The University of Melbourne Minerva database on the 21 February 2018 (http://hdl.handle.net/11343/198339). Ethics board approval was not required for this retrospective observational study. The applicable PRISMA guidelines [17] were adhered to.

**Fig 1:**
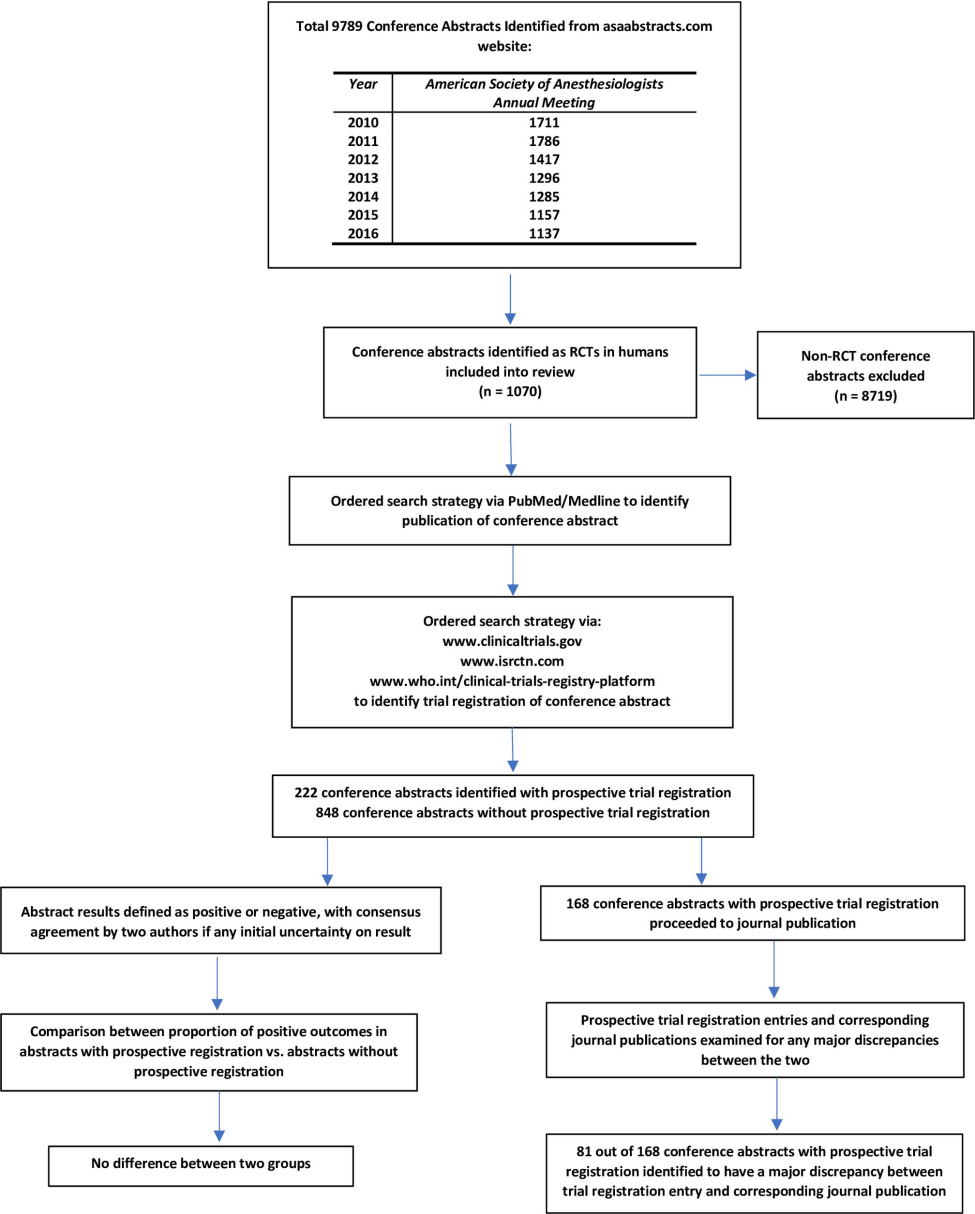
Flow diagram of review.

We conducted a review of all listed abstracts presented at the ASA Annual Meetings between 2010 and 2016 (Fig 1), using the ASA Abstracts website (www.asaabstracts.com). We used Microsoft Excel 2019 (Microsoft Corporation, Redmond, WA, USA) to construct a database including all abstracts that described RCTs in human participants. The timeline of 2010 to 2016 was selected for two reasons. First, this timeline allowed five years to have passed since the implementation of mandatory trial registration. Secondly, it allowed five years for abstracts to get published after being presented at the ASA meetings. When examining the data from our previous review [18], we noted that 94% (425 out of 454) of the positive abstracts and 92% (107 out of 117) of the negative abstracts reviewed that went on to publication, had been published by five years after being presented at the ASA meeting. We had no additional exclusion criteria.

Initially, as described prior in our protocol, abstracts were screened from ASA Meetings between 2010 to 2013. Fewer eligible abstracts were present in this timeframe than we had anticipated from our previous study [18] so abstracts from the ASA Meetings between 2014 to 2016 were included in order to meet our estimated required sample size.

An RCT was defined as a study in which prospective assignment of individuals via random allocation to one of two or more alternative healthcare interventions occurred [19]. An assessment of the primary outcome, or first reported outcome when there was no clear definition of the primary outcome, was made to determine if the abstract was “positive” or “negative”. Abstracts with a statistically significant result in favour of the experimental treatment for the primary outcome compared with the control treatment, were defined as positive. The exception to this was when the stated objective was to show treatment equivalence or non-inferiority. Studies that failed to show a statistically significant difference in the direction of the experimental treatment over the control treatment for the primary outcome, or failed to show equivalence or non-inferiority if that was the stated aim, were defined as negative. Any uncertainty over whether the primary outcome of the abstract was positive or negative was able to be resolved after two authors discussed and made a consensus decision guided by the above stipulated definitions.

We conducted a systematic search using PubMed and Medline to identify any subsequent publication of each included abstract. The search strategy included seven separate searches involving the use of the first author’s name, the second author’s name, the last author’s name, keywords, and a combination of different author’s names and keywords.

We then performed a systematic search for trial registration of all the RCT abstracts. If the trial registration number was available, this was used to examine the corresponding trial registry entry. If trial registration was not quoted in the abstract, we searched the clinicaltrials.gov website (United States Trial Registry website), the International Standard Randomised Controlled Trial Number Register (ISRCTN), and the World Health Organization International Clinical Trials Registry Platform. We searched the trial registry databases using multiple separate searches, again involving the use of the first author’s name, the second author’s name, the last author’s name, keywords, and a combination of different author’s names and keywords.

The following characteristics were recorded from each trial registration that was identified: date of registration, date of first patient enrolment or date of study commencement, primary and secondary outcomes, sample size estimation, study population, study intervention, inclusion and exclusion criteria, and country of origin. Trial registration was considered prospective when the date of registration was prior to the date of first patient enrolment. If the date of first patient enrolment was not specified, the date of study commencement (study start date) was used in its place.

The trial registration entries and corresponding journal publications were then examined for any major discrepancies. Major discrepancies were defined as primary outcome discrepancies, sample size changes of greater than 10%, or changes to the study intervention, without reporting or explanation of these changes. Primary outcome discrepancies included: the addition or omission of any primary outcome, changes in the definition of a primary outcome, or reclassification of a primary outcome to a secondary outcome and vice versa. As a secondary analysis, minor discrepancies were also examined, being defined as differences in secondary outcomes or inclusion and exclusion criteria, between the trial registration entries and corresponding journal publications.

The primary endpoint was a comparison between the proportion of positive outcomes in abstracts which had prospective registration and the proportion of positive outcomes in abstracts which did not have prospective registration. We defined a 20% difference in the proportion of positive outcomes to be of significant importance when comparing conference abstracts without prospective registration to conference abstracts with prospective registration.

### Sample size

Using the database from our previous review into publication bias in the anesthesia literature [18], it was noted that 1052 RCT abstracts were identified for inclusion from the ASA meetings between 2001 and 2004, of which 73% (n = 771) had positive outcomes. In addition, based on pilot data collected, the proportion of prospective trial registration was estimated to be approximately 20–25%. From this, a sample size of 223 in each group (total = 446) would have 90% power to show a 20% difference in the proportion of positive outcomes (73% versus 58%) overall.

### Statistical analysis

Statistical analyses were performed using Microsoft Excel 2019 (Microsoft Corporation, Redmond, WA, USA) and Stata version 16.1 (StataCorp. 2019. *Stata Statistical Software*: *Release 16*. College Station, TX: StataCorp LLC).

The proportion of abstracts with prospective trial registration was measured, along with the proportion of abstracts with prospective trial registration and a positive primary finding. A logistic regression model was fitted to compare studies with and without prospective trial registration. In addition, the proportion of prospectively registered trials with a major discrepancy between their trial registration entry and their corresponding journal publication was calculated. Finally, we quantified the types of major discrepancies that were found: primary outcome changes, sample size changes, or study intervention changes, and the types of minor discrepancies: secondary outcome changes and inclusion and exclusion criteria alterations.

To assess if the proportion of RCTs presented at the ASA meetings undergoing prospective trial registration increased over time, we fitted a general linear model with a logit link and binomial distribution, and used robust standard errors.

To estimate the association between prospective registration (yes/no) and publication (yes/no), we fitted a univariable logistic regression model. As a post hoc analysis, we also fitted a logistic regression model to estimate the association between registration (yes/no) and publication (yes/no) in the subgroup of abstracts with a positive primary finding.

## Results

From the ASA Meetings between 2010 to 2016, 9789 abstracts were presented and subsequently reviewed, with 1070 meeting our inclusion criteria as RCTs in humans.

Of the 1070 RCT abstracts, 222 (21%) underwent prospective trial registration. Of the abstracts that underwent prospective trial registration, 67% (149/222) had positive outcomes, whereas 73% (616/848) of those without prospective trial registration had positive outcomes (OR 0.77; 95% CI: 0.56 to 1.06; P = 0.105) (Table 1). Retrospective trial registration occurred in 268 out of 848 (32%) abstracts without prospective trial registration, i.e., trial registration occurred after the start of patient enrolment or study commencement date.

**Table 1:** Analysis of the relationship between prospective trial registration and study findings (positive or negative) of randomized controlled studies presented as abstracts at the American Society of Anesthesiologists Annual Meetings 2010–2016.

	*Negative conclusion (n = 305)*		*Positive conclusion (n = 765)*		*Univariable model*			
Prospectively registered	n	%	n	%	Odds ratio	95% CI - lower limit	95% CI - upper limit	p-value
**No**	232	76.1	616	80.5				
**Yes**	73	23.9	149	19.5	0.77	0.56	1.06	0.105

Overall, 52% of the 1070 abstracts progressed to journal publication within the five years after conference presentation. From the 222 abstracts that were prospectively registered, 168 (76%) went on to publication. In comparison, 386 of the 848 (46%) abstracts that did not undergo prospective registration, proceeded to publication. There was a strong association between abstracts that underwent prospective trial registration and proceeding to journal publication (OR 3.82; 95% CI 2.73 to 5.35; P < 0.001) (Table 2). This association remained strong when examining the subgroup of abstracts with a positive primary finding (OR 4.08; 95% CI 2.68 to 6.23; P < 0.001) (Table 3).

**Table 2:** Analysis of the relationship between prospective trial registration and publication outcomes (published or not published) of randomized controlled studies presented as abstracts at the American Society of Anesthesiologists Annual Meetings 2010–2016.

	*Not published*		*Published*		*Univariable model*	
Prospectively registered	n	%	n	%	Odds ratio (95% CI)	p-value
**No**	462	89.7	386	69.5		
**Yes**	54	10.3	168	30.5	3.82 (2.73, 5.35)	<0.001

**Table 3:** Analysis of the relationship between prospective trial registration and publication outcomes (published or not published) of randomized controlled studies with a positive primary finding, presented as abstracts at the American Society of Anesthesiologists Annual Meetings 2010–2016.

*Studies that had a positive primary outcome (n = 765)*						
Prospectively registered	Not published (n = 488)		Published (n = 564)		Univariable model	
	n	%	n	%	Odds ratio (95% CI)	p-value
**No**	325	52.8	291	47.2		
**Yes**	32	21.5	117	78.5	4.08 (2.68, 6.23)	<0.001

Of the 168 prospectively registered abstracts that went on to publication, 81 (48%) had a major discrepancy between their registry entry and corresponding published paper. The types and number of major discrepancies are displayed in Table 4. Minor discrepancies in secondary outcomes or inclusion and exclusion criteria were frequent (Table 4), with 109 (65%) secondary outcome discrepancies and 69 (41%) inclusion/exclusion criteria alterations noted.

**Table 4:** Breakdown of types of discrepancies between trial registration entries and corresponding journal publications.

	*Prospectively registered trials (N = 168) presented at the ASA Meetings 2010–2016 with corresponding journal publication for comparison with trial registry data*
**Major Discrepancy Type**	
Primary Outcome Downgrade or Upgrade	19
Primary Outcome Omission or Addition	23
Primary Outcome Change in Definition	30
Significant Sample Size Discrepancy	35
Intervention Group Change	11
Study Group Design Discrepancy	7
**Minor Discrepancy Type**	
Secondary Outcome Omission or Addition	89
Secondary Outcome Change in Definition	20
Inclusion/Exclusion Criteria Discrepancy	69

**Fig 2:**
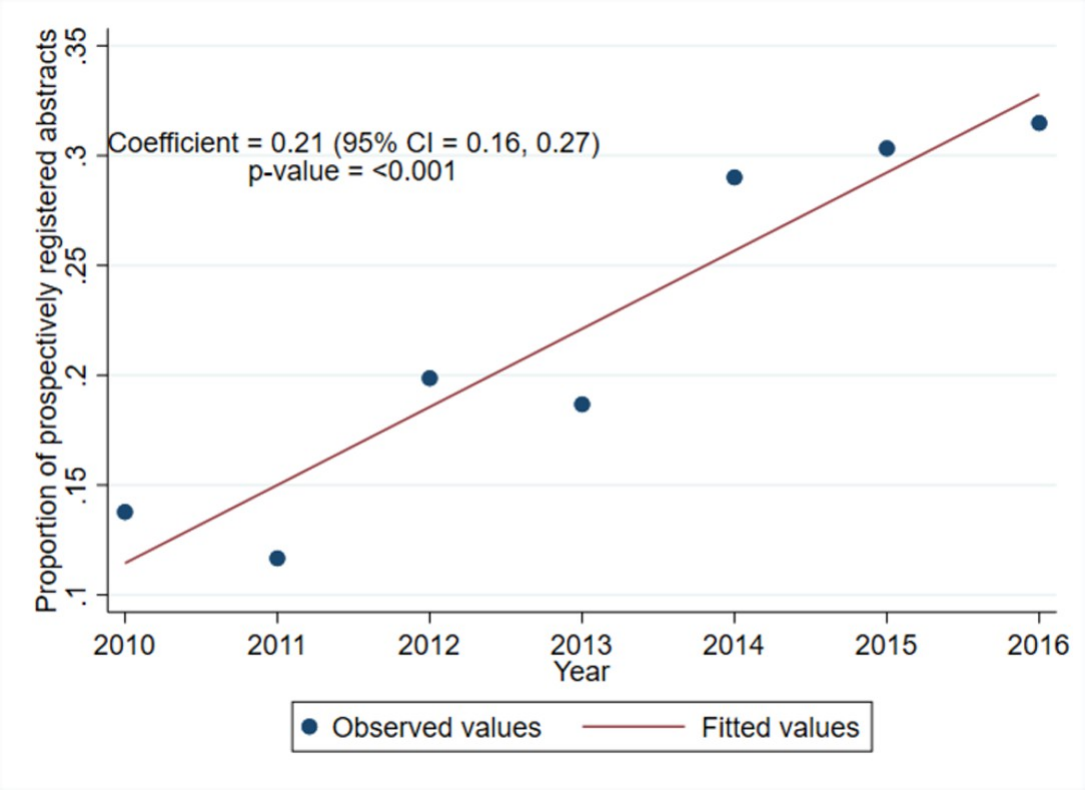
Proportion of Randomized-controlled trials presented at the American Society of Anesthesiologists Annual Meetings undergoing prospective trial registration over time.

Fig 2 displays a trend towards increasing proportions of RCTs presented at the ASA annual meetings undergoing prospective trial registration over the progressive years examined (mean change in proportion of prospectively registered abstracts per year 0.21; 95% CI: 0.16 to 0.27; P < 0.001).

## Discussion

Mandatory trial registration did not lead to a large proportion of trials being prospectively registered in anesthesia research during our seven year sampling period, although there was a progressive increase in the proportion of prospective registration during that time. Mandatory trial registration was implemented in 2005 [12]. In 1070 RCT abstracts presented between 2010 and 2016 at the largest annual scientific conference in anesthesia, only 21% of trials had been prospectively registered.

Moreover, prospective registration did not prevent researchers from altering their methods. From the abstracts with prospective trial registration, almost half (48%) had a major discrepancy between their trial registry entry and subsequent corresponding publication, demonstrating the presence of significant selective outcome reporting and associated bias. We found no evidence, however, for our hypothesis that there was a relationship between prospective registration non-compliance and reporting of positive trial outcomes.

Our results are consistent with previous studies. De Oliveira et al reviewed RCTs published in 2013 in the five highest impact factor anesthesia journals [15] and found that 64% of the trials were not prospectively registered and 48% of the registered trials had a major discrepancy compared with their trial registry entry. Jones et al reviewed RCTs published during four discrete years (2007, 2010, 2013, 2015) in high impact factor anesthesia journals, reporting that only 12% of the RCTs were adequately prospectively registered and 92% had an outcome discrepancy [16]. Notably, Jones et al employed a broader definition for discrepancies, defining this as at least 1 primary or secondary outcome discrepancy. The broader definition employed by Jones et al may have been due to the difference in populations examined, with their study sample focused solely on published RCTs, thus providing a potential explanation for their higher discrepancy rate of 92% [16].

De Oliveira et al. [15] and Jones et al. [16] both defined their ‘population’ of studies as RCTs published in high impact factor anesthesia journals. Our study methodology differs and expands on their work, by examining publication outcomes and selective outcome reporting earlier in the research and publication process, from the conference abstract presentation stage, through to the journal submission, review and publication endpoint. We found a strong association between prospective trial registration and progression to publication of RCTs presented as conference abstracts (OR 3.82; 95% CI 2.73 to 5.35; P < 0.001). Only 52% of abstracts achieved publication within the five year time window in our study, a similar proportion to that we found in our previous review of conference abstracts presented between 2001–2004 [18]. Given the substantial proportion of RCTs that go unpublished, prospective trial registration appears to be strongly associated with a successful publication outcome. This raises the question of whether this finding reflects a higher quality of trials that are prospectively registered, or higher rate of journal submission among prospectively registered RCTs, or a selective publication of prospectively registered RCTs by journals. In view of the low rate of prospectively registered abstracts and the equivalent abstract quality scores between published and unpublished abstracts demonstrated in our previous review [18], our finding supports the suggestion of selective publication by journals of prospectively registered trials.

Journal policies regarding trial registration do not protect against “retrospective trial registration”, whereby trials are registered after commencement of patient enrolment. The inability to prevent such retrospective data entry occurring is also a shortcoming of the clinical trial registry websites. In our sample, 268 abstracts (25%) underwent retrospective registration. Retrospective registration may conceal alterations to methods after commencement or completion of data collection, reducing the ability of registration to identify bias, and improved enforcement by journal editors is needed to reduce it.

Our study has several limitations. Consistent with Jones et al’s findings [16], our data collection revealed a significant decline in RCTs being presented over each successive year at the ASA meetings between 2010 to 2016. We initially planned to review four years of RCT abstracts from the ASA meetings between 2010 to 2013. The decline in RCT abstracts caused us to alter our study protocol, with an inclusion of three extra years, from 2014 to 2016, in order to achieve our desired sample size. As such, our overall sample of 222 abstracts that were prospectively registered and 848 abstracts that were not, provided greater power than originally planned. There was still a minimum period of five years post presentation, in order for publication to occur. Our previous review [18] found that at five years post abstract presentation, almost all studies that went on to subsequent publication, had been published (94% of positive abstracts and 92% of negative abstracts).

When evaluating for sample size discrepancies, we considered any change greater than 10% without explanation as a discrepancy. Some may argue the need for a more conservative definition, so we may have underestimated the presence of discrepancies. A further related limitation was that we did not explore the reasons why major discrepancies were occurring.

We also considered examining for discrepancies between the conference abstracts presented at the ASA meetings and their corresponding trial registration entries. However, a large proportion of the conference abstracts lacked sufficient detailed information on primary outcomes, study interventions and sample size calculations, rendering it difficult to make any meaningful comparison with the corresponding trial registry entries.

There are significant challenges to implementing a change such as the introduction of mandatory prospective trial registration. We have demonstrated that making prospective registration mandatory has not resulted in high rates of published studies actually having been prospectively registered in the anesthesia literature, and as demonstrated by related studies, in many other areas of medicine [20,21]. More time may still be required for prospective registration rates to increase, with the enforcement of trial registration by the major anesthesia journals having commenced over a wide range of dates from 2009–2015 onwards [16]. Our study supports this argument, as a post hoc analysis showed an increasing proportion of prospective trial registration by year, from 2010–2016 (Fig 2).

Even if adherence to mandatory prospective trial registration is improving, our findings reveal that almost half the studies that were prospectively registered still went on to have major discrepancies between their registry entries and corresponding journal publications. Until this issue is addressed, prospective registration cannot be considered as a solution for selective outcome reporting in the literature. A greater emphasis is still required for researchers to strive to undertake higher quality scientific work with the appropriate study design and power.

Our results, together with those of Jones et al. and De Oliveira et al. [15,16] have important clinical practice implications. The presence of significant selective outcome reporting, similar to positive publication bias, can spuriously skew bodies of evidence towards positive results, reducing the accuracy of summaries of that evidence. Benefits and treatment effects of interventions may be over-estimated by this bias and caution is necessary when interpretating published findings, particularly with small trials.

In conclusion, from a review of all RCTs presented at the ASA annual meetings from 2010 to 2016, we found a low proportion of prospective trial registration and a significant amount of major discrepancies between trial registry entries and corresponding journal publications. There was also a strong relationship between prospective trial registration and the likelihood of progression to journal publication. Improved enforcement of accurate and reliable prospective trial registration of RCTs is required. The contribution of non-compliance with prospective trial registration to selective outcome reporting in the anesthesia literature needs to be explicitly considered by reviewers, editors, and readers.

## Supporting information

S1 Checklist (DOCX)

## References

[1] Dickersin K, Chan S, Chalmers TC, Sacks HS, Smith H Jr. Publication bias and clinical trials. Control Clin Trials. 1987;8(4):343–53. doi: 10.1016/0197-2456(87)90155-3 3442991

[2] Dwan K, Altman DG, Arnaiz JA, Bloom J, Chan A-W, Cronin E, et al. Systematic Review of the Empirical Evidence of Study Publication Bias and Outcome Reporting Bias. PLOS ONE. 2008;3(8):e3081. doi: 10.1371/journal.pone.0003081 18769481 PMC2518111

[3] Dwan K, Gamble C, Williamson PR, Kirkham JJ. Systematic review of the empirical evidence of study publication bias and outcome reporting bias—an updated review. PLOS ONE. 2013;8(7):e66844. doi: 10.1371/journal.pone.0066844 23861749 PMC3702538

[4] Fleming PS, Koletsi D, Dwan K, Pandis N. Outcome discrepancies and selective reporting: impacting the leading journals? PLOS ONE. 2015;10(5):e0127495. doi: 10.1371/journal.pone.0127495 25996928 PMC4440809

[5] Hall R, de Antueno C, Webber A. Publication bias in the medical literature: a review by a Canadian Research Ethics Board. Can J Anaesth. 2007;54(5):380–8. doi: 10.1007/BF03022661 17470890

[6] Mathieu S, Boutron I, Moher D, Altman DG, Ravaud P. Comparison of registered and published primary outcomes in randomized controlled trials. JAMA. 2009;302(9):977–84. doi: 10.1001/jama.2009.1242 19724045

[7] Scherer RW, Langenberg P, von Elm E. Full publication of results initially presented in abstracts. Cochrane Database Syst Rev. 2007(2):MR000005. doi: 10.1002/14651858.MR000005.pub3 17443628

[8] Treanor L, Frank RA, Cherpak LA, Dehmoobad Sharifabadi A, Salameh JP, Hallgrimson Z, et al. Publication bias in diagnostic imaging: conference abstracts with positive conclusions are more likely to be published. Eur Radiol. 2020;30(5):2964–72. doi: 10.1007/s00330-019-06568-z 31953657

[9] Turner EH, Matthews AM, Linardatos E, Tell RA, Rosenthal R. Selective publication of antidepressant trials and its influence on apparent efficacy. N Engl J Med. 2008;358(3):252–60. doi: 10.1056/NEJMsa065779 18199864

[10] Walker KF, Stevenson G, Thornton JG. Discrepancies between registration and publication of randomised controlled trials: an observational study. JRSM open. 2014;5(5):2042533313517688. doi: 10.1177/2042533313517688 25057391 PMC4012655

[11] Glasziou P, Altman DG, Bossuyt P, Boutron I, Clarke M, Julious S, et al. Reducing waste from incomplete or unusable reports of biomedical research. The Lancet. 2014;383(9913):267–76. doi: 10.1016/S0140-6736(13)62228-X 24411647

[12] DeAngelis CD, Drazen JM, Frizelle FA, Haug C, Hoey J, Horton R, et al. Clinical trial registration: a statement from the International Committee of Medical Journal Editors. JAMA. 2004;292(11):1363–4. doi: 10.1001/jama.292.11.1363 15355936

[13] Wager E, Williams P. "Hardly worth the effort"? Medical journals’ policies and their editors’ and publishers’ views on trial registration and publication bias: quantitative and qualitative study. BMJ. 2013;347:f5248. doi: 10.1136/bmj.f5248 24014339 PMC3805489

[14] Chan A-W, Hróbjartsson A, Haahr MT, Gøtzsche PC, Altman DG. Empirical Evidence for Selective Reporting of Outcomes in Randomized TrialsComparison of Protocols to Published Articles. JAMA. 2004;291(20):2457–65. doi: 10.1001/jama.291.20.2457 15161896

[15] De Oliveira GS Jr., Jung MJ, McCarthy RJ. Discrepancies Between Randomized Controlled Trial Registry Entries and Content of Corresponding Manuscripts Reported in Anesthesiology Journals. Anesth Analg. 2015;121(4):1030–3. doi: 10.1213/ANE.0000000000000824 26378702

[16] Jones PM, Chow JTY, Arango MF, Fridfinnson JA, Gai N, Lam K, et al. Comparison of Registered and Reported Outcomes in Randomized Clinical Trials Published in Anesthesiology Journals. Anesth Analg. 2017;125(4):1292–300. doi: 10.1213/ANE.0000000000002272 28704247

[17] Page MJ, McKenzie JE, Bossuyt PM, Boutron I, Hoffmann TC, Mulrow CD, et al. The PRISMA 2020 statement: an updated guideline for reporting systematic reviews. BMJ. 2021;372:n71. doi: 10.1136/bmj.n71 33782057 PMC8005924

[18] Chong SW, Collins NF, Wu CY, Liskaser GM, Peyton PJ. The relationship between study findings and publication outcome in anesthesia research: a retrospective observational study examining publication bias. Can J Anaesth. 2016;63(6):682–90. doi: 10.1007/s12630-016-0631-0 27038290

[19] Lefebvre C, Manheimer E, Glanville J. Chapter 6: Searching for studies. In: Higgins J, Green S, editors. Cochrane Handbook for Systematic Reviews of Interventions. The Cochrane Collaboration 2011.

[20] McGee RG, Su M, Kelly PJ, Higgins GY, Craig JC, Webster AC. Trial registration and declaration of registration by authors of randomized controlled trials. Transplantation. 2011;92(10):1094–100. doi: 10.1097/TP.0b013e318232baf2 21978995

[21] Zarin DA, Tse T, Williams RJ, Rajakannan T. Update on Trial Registration 11 Years after the ICMJE Policy Was Established. N Engl J Med. 2017;376(4):383–91. doi: 10.1056/NEJMsr1601330 28121511 PMC5813248

